# Home Drug Delivery Service from the Perspective of Community Pharmacy Staff in Saudi Arabia

**DOI:** 10.3390/pharmacy10060162

**Published:** 2022-11-29

**Authors:** Majed Ahmed Algarni, Mona Yaser Alsheikh, Ahmed Ibrahim Fathelrahman, Meshal Suwaylih Alzaidi, Fawaz Jilan Faqeeh, Abdulelah Mohammad Althobaiti, Ali Mofleh Alshahrani

**Affiliations:** Department of Clinical Pharmacy, College of Pharmacy, Taif University, P.O. Box 11099, Taif 21944, Saudi Arabia

**Keywords:** community pharmacists, home drug delivery service, community pharmacy, access, healthcare services, equity

## Abstract

**Background**: In response to COVID-19, many big pharmacy chains in Saudi Arabia have started to provide home drug delivery services. This study aims to understand home drug delivery service from the perspective of community pharmacists in Saudi Arabia. Also, the study investigates the obstacles that may limit the use of home drug delivery service. **Methods**: A cross-sectional self-reported survey was distributed from February 2021 to May 2021. Descriptive analysis of sociodemographic characteristics was conducted and presented. Frequencies and percentages were calculated for all variables. **Results**: A total of 965 community pharmacists were surveyed. Most of the pharmacists, (73.5%) were young, aged 23 to 34 years old. The vast majority of the participants, (93.6%), said that the service will improve drug adherence. The lack of required knowledge and skills among pharmacists could be the main obstacle to implement home drug delivery service (34%). A shortage in the number of community pharmacists was the second main obstacle (24%). **Conclusion**: Home delivery services in the future may largely replace the tradition of going in person to the pharmacy. There are obstacles that may limit the full use of the service like shortage in number of pharmacists and the lack of required training.

## 1. Introduction

Until recently, dispensing medications was the dominant service provided by community pharmacies in Saudi Arabia [1]. However, the new Saudi government vision [2] and the need for other services are pushing community pharmacies to develop their services and to include new improved alternatives for patients, such as drug delivery services [3]. The need for and importance of home delivery services was substantially notable during the COVID-19 pandemic [4]. In Saudi Arabia, people could not get out of their homes due to the government-mandated quarantine that was issued for many months following the outbreak of the pandemic. Consequently, pharmacies were urged to provide home delivery services. The large chain pharmacies in Saudi Arabia started their home delivery services at that time and continued with the delivery service even when the quarantine was lifted. Public hospitals and health centers organized drug delivery services during the mandated quarantine. Patients need home care services not only in times of pandemics and quarantines but also in normal situations, especially, geriatric patients, patients with disabilities, or those with chronic illnesses [5]. Pharmaceutical delivery services may facilitate patient accessibility to medications, ensuring drug adherence, which in turn improve public health and the whole healthcare system [6]. The role of the pharmacist has traditionally been restricted to prepare, and dispense medicines [7], however, the pharmacy profession has evolved in the last few decades to improve patient outcomes and contribute to the advancement of health care system. In developing countries such as Saudi Arabia, national efforts are often directed towards ensuring the availability of and accessibility of drugs for all patients [8]. Nevertheless, community pharmacy roles are expanding in Saudi Arabia, and the new governmental vision is accelerating the expansion process [2]. Examples of expanded roles established recently in community pharmacies are the provision of COVID-19 vaccines and the management of patients with chronic diseases. Drug delivery services have been used in many countries for years, and have been a helpful tool in reducing the costs. For instance, in the USA, the mail service helped to decrease the total costs of prescriptions [9,10]. Recently, in Saudi Arabia, the Saudi Postal Services & Logistics (SPL) and the Ministry of Health (MOH) signed an agreement to deliver medications to patients who are treated in MOH hospitals and health centers at their homes, where the agreement aims to cover 284 hospitals and medical centers in various regions [11]. To date, a million drug deliveries have been made. The beneficiaries are normally patients receiving refills and when new patients are included in the services they get an appointment with a virtual pharmacy-led clinic to receive the required education before being covered by the medication delivery services. On the other hand, Alnahdi Medical Company, a private operator of one of the largest series of community pharmacies in Saudi Arabia, adopted three packages of services, with one for home medication delivery, one for medication adherence, and another for elderly patients where nurses and other healthcare providers provide home care for the elderly patients with chronic diseases. Pharmacists are involved directly in the medication adherence services by providing counseling and performing assessment of adherence among patients. The delivery service is normally provided separately to the pharmacy, but occasionally pharmacy technicians may be involved in the delivery process. In this study, our objective is to comprehend and highlight the understanding and attitudes of pharmacists in Saudi Arabia regarding drug delivery services. Since pharmacists are the ones who dispense medications for patients, educate patients about their medications, and communicate with patients in many levels, it is imperative to know more about their view and understanding regarding drug delivery service.

## 2. Materials and Methods

We implemented a cross-sectional self-reported survey to assess drug delivery services provided by community pharmacy staff. The survey questions were specifically designed for the purpose of the current study. Two staff from College of Pharmacy, Taif University, with good practice- and research-based experiences validated the questionnaire. The reviewer feedback was used to edit the survey as needed. A pilot sample from community pharmacy staff was used to test the survey’s appropriateness, including easiness, clarity, and the required time for completion. We adopted a convenience sampling technique to recruit community pharmacy staff between February 2021 and May 2021. In Saudi Arabia, there were 8419 community pharmacists out of 24,395 licensed pharmacists (34.5%) working in different pharmacy sectors [12]. For this study, it was determined that surveying at least 842 pharmacy staff from different regions across Saudi Arabia would be representative (i.e., 10%). The self-reported survey was distributed to both individual pharmacies and to groups of chain pharmacies. The link to the survey was delivered via social media such as WhatsApp groups and Twitter. Some keypersons facilitated the communication with participants. All participants were provided with informed consent. In line with the ethical research requirements guiding this study, all data were collected anonymously with no personally identifiable information about the participants being collected. An approval from the Scientific Research Ethics Committee at Taif University was obtained with the number HAO-02-T-105. The survey was divided into two sections. The first section was about the demographic profile for the community pharmacist, for example, gender, age, educational level, monthly income, the region the pharmacist lives in, professional classification. The survey tool was used for assessing drug delivery services from the perspective of community pharmacists. Descriptive analysis of sociodemographic characteristics is conducted and presented here. Frequencies and percentages were calculated for all variables. Microsoft Excel^®^ version 16.53 was used to clean, sort, and analyze the data.

## 3. Results

A total of 965 licensed pharmacy staff participated in the survey. The participants were from all different regions of Saudi Arabia. Most of them (88.5%, *n* = 854) were male, whereas only (11.5%, *n* = 111) were female. Most of participants were aged 23–34 years old (73.6%), followed by those aged 35–44 years old (22.2%). Only a fourth of the sample were Saudis (*n* = 239, 24.7%), while 726 were non-Saudi (75.2%). Respondents from the western region of Saudi Arabia represents the majority with 401 participants (41.5%), while the eastern region was the least represented by only 81 participants (8%). Most of the participants (*n* = 800, 82.9%) were holding a Bachelor’s degree, 127 with a PharmD (13%), and only 27 have a MSc or PhD (2.7%). By professional classification (i.e., according to Saudi Commission for Health Specialties), the highest proportion were pharmacists (70.2%), followed by senior pharmacists (25.6%), consultant pharmacists (3.2%), and finally, pharmacy technicians (1.1%). The vast majority of pharmacy staff work in chain pharmacies (*n* = 895, 92.7%), and few work in independent pharmacies (7%). Only 101 out of 965 participants said they are not satisfied with their job (10%). About two thirds (*n* = 616, 64%) have an experience for more than 5 years. Table 1 below provides a summary of the demographics of the participants.

**Figure 1:**
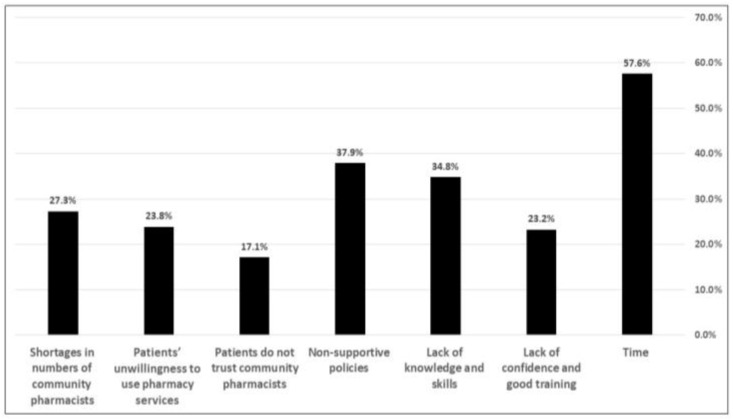
Obstacles that may limit implementing home drug delivery services in Saudi Arabia.

Most of the surveyed community pharmacy staff (*n* = 689, 71.4%) agreed that drug delivery helps patients to continue taking their medication, while only 61 (6%) disagreed about that (Table 2). Out of 965 surveyed pharmacists, 91% (*n* = 879) think that pharmacists can provide remote consultation if there is a consulting application, while the other 9% (*n* = 86) disagreed about that. More than half of the surveyed pharmacists (61%, *n* = 586) also agreed that pharmacists can monitor the health of patients with chronic conditions in their homes, while 39% (*n* = 379) disagreed. Most of the surveyed pharmacy staff (81.4%, *n* = 786) believe that delivery apps would become a substitute for going to the hospital to collect treatment, whereas the rest (18.5%, *n* = 179) did not believe that drug delivery would substitute going to a hospital for collecting medications (Table 2). Around 73% (702 out of 965) of the surveyed respondents chose respiratory system diseases to be among the most diseases that pharmacists need to be educated about for drug delivery service (Table 2). Of the surveyed pharmacists, 884 community pharmacists preferred to sell medications directly inside the pharmacy (92%), while 81 pharmacists preferred to sell them online (8%). The highest proportion (*n* = 688, 71.3%) of the surveyed respondents think that pharmacists are the most qualified to deliver home drug delivery. When asked whether they support pharmacist to charge a fee for providing delivery services, 72% (*n* = 689) of the respondents said “yes”, while 28% (*n* = 267) chose “no” as answer for that question (Table 2). The surveyed community pharmacists when asked about the obstacles that may limit implementing home drug delivery service, 57.6% chose time, 34.8% chose the lack of the required knowledge and skills, 27.3% chose that shortage in number of community pharmacists could be the main obstacle, 37.9% said that the lack of coordinated and supportive policies might be the main obstacle, 23.8% said that patients’ unwillingness to use home drug delivery service could be the main obstacle, and 23.2% said that lack of required pharmacist’ confidence and good training is the main obstacle (Figure 1).

## 4. Discussion

Community pharmacy roles have increased in the last few decades. They have become more than a dispensing place, also a substantial player in the health care system, with a range of different services. Among the services adopted by community pharmacies recently, in Saudi Arabia, delivery services are particularly clinically oriented services that help with ensuring patient health outcomes. To the best of our knowledge, this is the first study that has studied drug delivery service from the perspective of community pharmacists in Saudi Arabia. In total, 965 community pharmacists participated in this study. Most of the participating pharmacists agreed that drug delivery services would improve patient drug adherence. This result coincides with many previous published studies that were done in countries with drug delivery service, in which, the results showed that delivery service increased drug adherence for treating chronic diseases like diabetes [13,14,15,16,17]. As a logistical service, drug delivery helps patients receive their medication on time, thus, to maintain treatment and have better health indicators. The majority of the surveyed community pharmacists (91%) agreed that proper and necessary consultation could be done remotely. This result matched with many published articles that approved both the importance and the success of remote consultation, especially in pandemic situations like COVID-19, where the most precautionary measures should be taken [18,19,20]. Moreover, 81% of the surveyed pharmacists agreed that home delivery services would largely replace the old tradition of going in person to a pharmacy to receive or refill the prescribed medications. Findings in a recently published article showed that home delivery services and telepharmacies are some of the most important factors that represent a major shift in pharmacy practice, especially in the context of COVID-19 [21]. Further, a study that was conducted in Spain found that the majority of patients used home delivery services since the COVID-19 pandemic started [22]. Studies conducted in the eastern region of Saudi Arabia found that ambulatory pharmacy services were well sustained via home delivery services [23,24]. This shift of pharmacy practice toward home delivery and telepharmacy care may not get slower, even if the COVID-19 pandemic is resolved, since most people, especially elderly patients or those with special needs, or even normal people, may depend more and more on such services, since it is easier and more comfortable than the traditional service. This can be viewed as an innovation and a positive change in pharmacy practice; however, it also represents a challenge to pharmacy personnel in the future and the pharmacy profession should keep advancing its provided services to stay at the frontlines of healthcare. Literature from the Arabic region has highlighted the pros and cons of home drug delivery from the perspectives of both pharmacists and the public. In Jordan, three thirds of community pharmacists surveyed supported the use of home medication delivery [25]. However, half of the respondents in this study think that such a service is suitable for refill prescriptions and not for new prescriptions. One of the benefits identified by pharmacists was that these services decrease congestion at health facilities. Another study from Jordan revealed supportive findings among the public [26]. Participants tend to believe that home delivery of medication is more convenient and accessible than in-store drug refills and most of them believed that such services are more suitable for refill-prescription drugs. A study from Lebanon investigated perceptions of the public regarding home medication delivery [27]. Participants thought that home delivery would negatively affect pharmaceutical care since it makes pharmacists less accessible for answering patient questions and prevents pharmacists from explaining important information about medications. On the other hand, as expected, the majority of the surveyed community pharmacists in our study (72%) said that those pharmacists who prepare and supervise delivery services deserve an extra fee for their services. Only 28% indicated that it is not necessary to have an extra fee. A considerable portion of the surveyed pharmacists (19%) considered that a lack of coordinated and supportive polices is one of the main challenges that may hinder home delivery services. Two recently published articles coincide with such results, which agree that excellent communication and coordination between the different involved sectors in home delivery services is a key factor in ensuring a good service [28,29]. Another 10% of the participated pharmacists said that lack of appropriate training, like communication skills, is the main obstacle, which also coincides with previous published studies’ results [28,29]. Females represented a small proportion of participants. However, according to previous reports this low representation reflects the actual situation of practice at community pharmacy at the time of study. For example, AlRuthia et al., reported that females represented only 14.84% of licensed pharmacy workforce working in different settings of practice in Saudi Arabia [12]. Knowing that the community pharmacy was not the preferred setting for practice among female pharmacists, most were working in hospitals, industry, and regulatory sectors. Almaghaslah and associates also reported that community pharmacists constitute 74.5% of the pharmacy workforce in the private sector in Saudi Arabia and that females account for 0.3% [30]. To our knowledge, this is the first study documenting home drug delivery in Saudi Arabia on a national scope. The strengths of the present study are the relatively large sample size and presence of representation from all regions across Saudi Arabia. A limitation of study was that the regions of Saudi Arabia were not equally represented in the study, with the majority of pharmacists being located in the western region. Another limitation is that rural pharmacists were a minority in the study. This study addressed home drug delivery services from the perspective of community pharmacy staff. The findings represent how respondents perceive the importance and value of such services and do not assess the actual practice. However, they are a good baseline for future studies on the topic.

Improving health care systems to provide high quality and cost-effective healthcare services to treat a needy population could be a challenge, and home drug delivery services could play a part to improve such a healthcare system. Previous studies have shown that home delivery services provided by community pharmacies may play a role in decreasing the costs of the health system, improving medication management, reducing emergency department visits and hospital admissions, and improving the overall quality of life.

## 5. Conclusions

This study shows that community pharmacy staff believe that drug delivery services will improve medication compliance. Respondents who participated in this study gave a clear vision about their expectations about home delivery services provided by community pharmacies in Saudi Arabia. In the future, home delivery services may largely replace the tradition of in-person pharmacy visits. However, according to the surveyed community pharmacists, there are obstacles that limit the full use of such services, like a shortage in number of pharmacists and the lack of required training. These obstacles should be eliminated to allow the full benefit of home drug delivery services.

## Figures and Tables

**Table 1:** Demographic characteristics of participated community pharmacy staff.

Variable	Frequency (No.)	Percent (%)
**Age (years)**		
23–34	710	73.6
35–44	214	22.2
45–54	33	3.4
55–60	8	0.8
**Gender**		
Male	854	88.5
Female	111	11.5
**Nationality**		
Saudi	239	24.8
Non-Saudi	726	75.2
**Region**		
Southern region	171	17.7
Eastern region	81	8.4
Western region	401	41.6
Northern region	122	12.6
Central region	190	19.7
41.6% of the respondents reside in the		
**Educational level**		
Diploma	12	1.2
Bachelor	800	82.9
Pharm.D	127	13.1
Master or PhD	27	2.7
**Professional classification**		
Consultant pharmacists	31	3.2
Senior pharmacists	247	25.6
Pharmacists	677	70.2
Pharmacy technician	10	1.1
**Years of experience in the pharmacy work**		
<2 years	160	16.6
2–5	189	19.6
6–10	360	37.3
>10 years	256	26.5

**Table 2:** Opinions and perceptions of community pharmacy staff towards home drug delivery.

Questions	Yes (%)	No (%)
**If there is a consulting application, can the pharmacist provide remote consultations?**	879 (91%)	86 (9%)
**Can the pharmacist monitor the health of patients with chronic diseases in their homes?**	586 (61%)	379 (39%)
**Do you support the pharmacist to charge a fee for providing the pharmaceutical services delivery service?**	689 (72%)	267 (28%)
**Does drug delivery help patients to continue taking their medication?**		
Strongly disagree	24 (2.5%)	
Disagree	37 (3.8%)	
Neutral	215 (22.3%)	
Agree	414 (42.9%)	
Strongly agree	275 (28.5%)	
**Are delivery apps a substitute for going to the hospital to collect treatment?**		
Never	123 (12.7%)	
Rarely	56 (5.8%)	
Sometimes	507 (52.5%)	
Often	225 (23.3%)	
Always	54 (5.6%)	
**Where do you prefer to Sale medicines?**		
Online	81 (8%)	
In Pharmacy	884 (92%)	
**Who is the person qualified to provide and deliver pharmaceutical services? (*Respondents may choose more than one answer*)**		
Consultant pharmacists	287 (29.7%)	
Senior pharmacists	342 (35.4%)	
Pharmacists	688 (71.3%)	
Pharmacy technicians	385 (39.9%)	
**What are the most diseases that a pharmacist needs to be educated about when caring for patients? (*Respondents may choose more than one answer*)**		
Respiratory diseases	702 (72.7%)	
Infectious diseases	688 (71.3%)	
Heart diseases	618 (64.0%)	
Liver and kidney disease	441 (45.7%)	
Endocrine disease	438 (45.4%)	
Psychological disease	434 (44.9%)	
Blood diseases	400 (41.4%)	

## Data Availability

Data are available upon reasonable request to the corresponding author.
